# Thromboembolic prevention in athletes: management of anticoagulation in sports players affected by atrial fibrillation

**DOI:** 10.3389/fphar.2024.1384213

**Published:** 2024-05-10

**Authors:** Simona Minardi, Luigi Sciarra, Antonio Gianluca Robles, Antonio Scara, Francesco Sciarra, Gabriele De Masi De Luca, Silvio Romano

**Affiliations:** ^1^ Department of Clinical Medicine, Public Health, Life and Environmental Sciences, University of L’Aquila, L’Aquila, Italy; ^2^ Department of Cardiology, L. Bonomo Hospital, Andria, Italy; ^3^ Department of Cardiology, San Carlo di Nancy-GVM, Rome, Italy; ^4^ La Sapienza University, Rome, Italy; ^5^ Department of Cardiology, Card. G. Panico Hospital, Tricase, Italy

**Keywords:** atrial fibrillation, anticoagulation, sport cardiology, antithrombotic therapy, athletes

## Abstract

Atrial fibrillation (AF) is a common cardiac arrhythmia that poses a significant risk of stroke and thromboembolic events. Anticoagulation therapy is essential for preventing stroke in patients with AF. An increasing number of people of all ages, including cardiac patients, approach physical activity as both a leisure-time exercise and a competitive sport. Therefore, patients at risk of AF are increasingly allowed to practice sports activities. Management of oral anticoagulant therapy (OAT) in these patients is extremely challenging because of the need to balance the risks and benefits of medications, considering both hemorrhagic (in case of trauma) and ischemic complications when the drugs are avoided. Official recommendations are limited for these patients and forbid sports that increase the risk of trauma and consequent bleeding in most cases. These recommendations are strongly influenced by the “traditional” management of OAT, which mainly involves coumarin derivatives. Non-vitamin K antagonist direct oral anticoagulants (DOACs), with their more favorable pharmacokinetic–pharmacodynamic profile than that of coumarin derivatives, may represent an opportunity to modify the approach to sports activity in patients with AF and indications for OAT. This study aimed to review the use of anticoagulants in athletes with AF, highlight their efficacy and safety, and provide practical considerations regarding their management.

## 1 Introduction

Atrial fibrillation (AF) is one of the most frequent cardiac arrhythmias, with an estimated prevalence of 2%–4% in the general population (Hindricks et al., 2020). The conventional risk factors include hypertension, diabetes, obesity, coronary artery disease, and valvular disease. Regular physical activity at moderate intensity is a protective factor that is recommended for prevention of AF (Mozaffarian et al., 2008) and other cardiovascular diseases. Exercise programs are part of optimal therapy and are prescribed in association with drugs by cardiologists (Mezzani et al., 2013). Physical activity has consequently spread among people of all ages and the phenomenon is constantly expanding (Lee et al., 2012). Therefore, an increasing number of athletes are “masters,” i.e., older than 35 years, and elderly and have a higher probability of AF associated with age and comorbidity.

The main risk factors for AF are stroke and thromboembolic events, for which strict oral anticoagulation therapy (OAT) is required in patients with thromboembolic risk factors (Hindricks et al., 2020). Elderly athletes usually have thromboembolic scores that indicate the need for anticoagulation therapy. Definitive data on the prevalence of permanent OAT in the sports population are lacking. However, the problem is negligible when we consider that these patients, especially those who perform sports associated with a high risk of trauma, face a unique challenge with anticoagulation therapy because of the need to balance thromboembolic prevention and hemorrhagic risk (Abdulla and Nielsen, 2009). The absence of specific randomized trials with this cohort of patients makes decision-making regarding anticoagulation management based on observational data and expert consensus particularly challenging. Current evidence suggests the same indication for athletes as for the general population, i.e., anticoagulant therapy for all patients with AF who have a high thromboembolic risk, based on a high CHA_2_DS_2_-VASc score (Lip et al., 2010; Kirchhof et al., 2013; Kirchhof et al., 2016; Steffel et al., 2018; Palamà et al., 2023). The problem with athletes treated with anticoagulants is related to the risk of traumatic events that burden every sports activity, despite the large variability related to a specific discipline. In light of this evidence, participation in contact and collision sports is not recommended, whereas non-contact sports are considered safe without the need for anticoagulation interruption (Maron et al., 2015). Most recommendations are based on anticoagulation therapy with coumarin derivatives. Currently, direct oral anticoagulants (DOACs), with their short half-lives, have substantially changed the management of AF and are suitable alternatives to coumarin derivatives in stroke prevention, especially in patients with an elevated bleeding risk (Browne et al., 2018; Steffel et al., 2018), as found in sports practice. However, its potential implications for athletes remain largely unexplored in many registries. DOACs introduce the possibility of a “therapeutic window,” outside which, when the effect of anticoagulants is over or minimal, some contact sports could be allowed in appropriate situations (Romano et al., 2020). This review focuses on the existing evidence on OAT in people with AF practicing sports activities and on perspectives in the DOAC era.

## 2 Prevalence of AF in athletes

AF is the most common sustained cardiac arrhythmia, affecting 2%–4% of the general population (Hindricks et al., 2020), and this percentage is likely to increase 2–5-fold over the next 50 years, reflecting the growing proportion of elderly individuals (Go et al., 2001). Therefore, this constitutes a relevant issue for increasingly older people practicing sports activities. The prevalence of AF in athletes ranges from 0.3% to 12.8% (Pelliccia et al., 2005a; Wernhart and Halle, 2015; Calvo et al., 2016; Sanchis-Gomar et al., 2017; Sharma, 2018; Newman et al., 2021), depending on the age group and the type of sports analyzed. The main predisposing condition is age. Indeed, Wernhart and Halle (2015) observed that the prevalence of AF in younger athletes is very low and irrelevant, while arrhythmia is as common in athletes aged > 65 years as in the general population since it is likely caused by structural heart disease. The lowest prevalence was reported by Pelliccia et al. (2005a), who enrolled 1,777 athletes aged 24 ± 6 years and reported symptomatic paroxysmal AF in only 0.3% of them. Karjalainen et al. (1998) analyzed AF in middle-aged (47.5 ± 7 years) athletes and found a mild prevalence of 5.3%. Grimsmo et al. (2010) examined 149 healthy endurance athletes aged 58.9–88.5 years and found a high prevalence of AF (12.8%). Sharma et al. used electrocardiogram (ECG) to screen marathon runners aged > 35 years and revealed that 5%–10% experienced AF (Sharma, 2018). Most previous studies enrolled healthy patients practicing sports but reported limited data on CHA_2_DS_2_-VASc scores; therefore, their thromboembolic risk was assumed to be low and increased with age. Considering the growing number of elderly athletes who usually have high thromboembolic scores, the indications for antithrombotic therapy in athletes are expected to increase. In 2016, Myrstad et al. (2016) enrolled 4,952 Norwegian men and women aged 53–85 years, 2,626 veteran cross-country skiers, and 2,326 members of the general population. Data on physical activity, endurance exercise, functional capacity, comorbidities, drug use, subjective health, and AF were collected using questionnaires. AF was self-reported and confirmed using electrocardiography in a review of medical records. The prevalence of self-reported AF among veteran skiers was 12.3%, whereas that of AF was 5.3%. Interestingly, many veteran skiers with AF were not optimally treated with OAT; indeed, only two out of three with CHA_2_DS_2_-VASc scores ≥2 used anticoagulants, and 11% stroke prevalence was observed (Myrstad et al., 2016).

These observations indicate that, as in the general population, the incidence of AF in athletes increases with age. Studies have observed that the prevalence of AF in athletes is 2–10 times greater than that in the general population (Sharma, 2018). This finding suggests a U-shaped relationship between sports and AF (Aizer et al., 2009; Andersen et al., 2013; La Gerche and Schmied, 2013; Morseth et al., 2016; Sciarra et al., 2022; Petrungaro et al., 2023), whereby AF seems to increase due to either deficient or excessive sports practice, and patients instead benefit from moderate exercise. The assumed mechanisms underlying this observation include fibrosis or inflammation, hemodynamic variations leading to left atrial enlargement, vagal tone, and genetic factors triggered by physical exercise (Flannery et al., 2017). Moreover, some hypothesize that endurance or mixed sports and >2,000 h of total lifetime exercise are independent risk factors (Calvo et al., 2016). A systematic review by Newman et al. (2021) observed no significant difference in the risk of AF between athletes and non-athletes with cardiovascular disease (CVD) risk factors. When CVD was excluded from the analyses, the risk of AF in athletes was significantly higher than that in non-athletes. Younger athletes have a higher incidence of AF than controls, as demonstrated in meta-analyses by Ayinde et al. (2018) and Newman et al. (2021) However, this population also has a low thromboembolic risk score and does not require OAT. Specific data on the AF burden in athletes are lacking; therefore, future prospective studies for evaluating these aspects are needed. In summary, the prevalence of AF increases with age; therefore, the impact of AF on athletes is substantial, owing to the increase in the number of elderly people with CVD risk factors practicing sports. Consequently, the management of OAT in patients practicing sports activities will become a growing problem in the coming years.

## 3 Anticoagulation indications and options

Indications for OAT in patients with AF are driven by thromboembolic risk factors (Hart et al., 2007; January et al., 2019; Hindricks et al., 2020). The most common tool used in clinical practice to determine the risk of cardioembolism is the CHA_2_DS_2_-VASc score, which is calculated by assigning one point to congestive heart failure (C), hypertension (H), diabetes (D), vascular disease (V), an age of 65–74 years (A), and sex category (Sc) and two points to an age of ≥75 years (A2), previous stroke, transient ischemic attack, or thromboembolism (S2) (Lip et al., 2010; Hindricks et al., 2020). Multiple studies and international guidelines recommend OAT when AF occurs in men with CHA_2_DS_2_-VASc scores ≥2 or in women with CHA_2_DS_2_-VASc scores ≥3^1^. Men with CHA_2_DS_2_-VASc scores of 1 and women with CHA_2_DS_2_-VASc scores of 2 should be considered for OAT, considering additional factors, such as bleeding risk and patient preference (Chao et al., 2015). Male patients without thromboembolic risk factors or female patients with CHA_2_DS_2_-VASc scores of 1 (i.e., no risk factors other than sex) are not recommended for OAT, except for a short period of anticoagulation required in the case of cardioversion or catheter ablation procedures (Hindricks et al., 2020).

Until recently, the cornerstone of OAT was a class of drugs whose mode of action was to reduce blood levels of coagulation factors II, VII, IX, and X by inhibiting the hepatic vitamin K-dependent protein post-translation modifier system.

Since 2010, when the first DOAC became clinically available in the USA, this class of drugs has been preferred because of its advantages over coumarin derivatives, including ease of use, a wider therapeutic range with less strict monitoring requirements, fewer drug interactions, and a favorable pharmacological profile with a fixed dose (Biswas et al., 2023). The pharmacokinetic and pharmacodynamic properties of DOACs and coumarin derivatives are summarized in Table 1.

**TABLE 1:** Pharmacokinetic and pharmacodynamic properties of DOACs and coumarin derivatives (Wang and Bajorek, 2014; Steffel et al., 2021).

	Dabigatran	Apixaban	Edoxaban	Rivaroxaban	Coumarin derivatives
Target	Thrombin	fXa	fXa	fXa	Vit K epoxide reductase (VKORC1). Lower levels of vit-K-dependent factors (II, VII, IX, and X)
Prodrug	Yes	No	No	No	No
Reversible inhibition	Yes	Yes	Yes	Yes	No
Bioavailability	3%–7%	50%	62%	66%; 80%–100% with food intake	>95%
Food intake effect	Delayed, no reduction in bioavailability	None	None	Augmented bioavailability	None
Distribution volume (L)	60–70	21	>300	50	10
Protein binding	35%	87%	40%–59%	>90%	99%
Other carriers	Unknown	BCRP/ABCG2	Unknown	BCRP/ABCG2	None
Cmax (H)	1–3	3–4	2	2–4	2–4
Half-life (H)	12–17	12	10–14	5–9	40
Cytochrome metabolism	None	CYP3A4/5, CYP2J2 (minor), and CYP1A2 (minor)	CYP34A	CYP3A4/5 and CYP2J2 (equal)	CYP2C9, CYP3A4, CYP2C19, and CYP1A2
P-GP substrate	No	Yes	Yes	Yes	No
Renal clearance/non renal clearance of the absorbed dose	80%/20%	27%/73%	50%/50%	35/65%	Metabolites eliminated 80%/20%
Hemodialysis clearance	60%–70%	Unlikely	Possible	Unlikely	No
Daily administration frequency	Twice	Twice	Once	Once	Once
Formulation	Capsule (unopened and uncrushed)	Pill (crushable)	Pill (crushable)	Pill (crushable)	Pill (crushable)
AF stroke prevention standard dose	150 or 110 mg/bid	5 mg/bid	60 mg/day	20 mg/day	Variable according to INR
Lower dose	110 mg/day	2.5 mg/bid	30 mg/day	15 mg/day	
Dose-reduction criteria	• ≥80 years old or • concomitant therapy with a strong P-GP inhibitor or • increased bleeding risk	• CrCl 15–29 mL/min or if 2 of: • weight <60 kg • Cr > 1.5 mg/dL • >80 years of age	• weight <60 kg or • CrCl <50 mL/min or • concomitant therapy with a strong P-GP inhibitor	CrCl <50 mL/min	

AF, atrial fibrillation; Cr, creatinine; CrCl, creatinine clearance; P-GP, P-glycoprotein.

Athletes, like the general population, can benefit from these agents for stroke prevention. Moreover, DOACs could be ideal anticoagulants for athletes because of the possibility of strategic planning of drug administration, and thus, in appropriate situations, allowing even some contact sports, limited to the timeframe in which the anticoagulant effect is over or considered minimal (Romano et al., 2020). However, the pattern of drug elimination from blood may vary depending on single DOAC, and their residual activity (DOAC concentration and anti-factor Χa activity) may not be negligible, even 24 h after the last intake, as observed by Sairaku et al. (2019)

## 4 OAT in athletes: guideline recommendations and possible management

Studies of OAT in athletes have been limited and mainly based on the clinical experience of patients treated with coumarin derivatives. The indications for OAT during AF are similar for athletes and the general population and could differ from the management of therapy in these patients. Indeed, the greatest risk for people taking OAT is bleeding in the event of injury, and athletes are often at an increased risk of trauma compared to the general population. In 2022, Chandran et al. (2022) conducted an epidemiological study examining sports-related concussions (SRCs) in 23 National Collegiate Athletic Association (NCAA) sports from 2014/2015 to 2018/2019. Of the 8,474,400 athlete exposures (AEs), 3,496 SRCs were reported; the competition-related SRC rate was higher than the practice-related SRC rate (IRR: 4.12; 95% CI: 3.86–4.41), men’s ice hockey and women’s soccer had the highest SRC rates (7.35 and 7.15 per 10,000 AEs, respectively), and the most frequently reported mechanism was player contact in men’s sports (77.0%) and equipment/apparatus contact in women’s sports (39.2%). Similarly, a recent analysis by Stewart and Guseh, 2023) identified American football, ice hockey, soccer, basketball, and lacrosse as sports that are most likely to increase bleeding risk in athletes.

Every sport is burdened with the risk of trauma, although it varies significantly between disciplines. According to this risk, sports can be categorized as contact, limited contact, or non-contact (Rice, 2008). This classification has been performed for pediatric athletes but can also be extended to adults. Non-contact sports such as running and swimming are characterized by a low probability of trauma, which is usually inadvertent and unexpected. Sports such as baseball and volleyball are considered limited-contact sports because contact remains unintentional and infrequent. In contact sports such as basketball and soccer, a certain degree of trauma recurrently occurs. Furthermore, collision sports such as American football and ice hockey have significant bodily contact as the main characteristic of the activity.

The 36th Bethesda Conference (Heidbüchel et al., 2006) and international guidelines^1,11,42.43^ recommend that sports with direct bodily contact or a risk of trauma should be avoided in patients undergoing OAT. European guidelines (Hindricks et al., 2020; Pelliccia et al., 2020) and consensus documents (Heidbüchel et al., 2006) recommend participation in sports activities for asymptomatic AF patients without structural disease if adequate rate control during exercise is achieved; however, no contact sports for athletes on OAT are allowed (Class III; Level A). Similarly, the American Heart Association and American College of Cardiology (Maron et al., 2015) recommend that athletes with a history of AF receiving long-term OAT should not engage in sports involving any risk of bodily contact because of the increased risk of intracranial hemorrhage (Class III; Level C). The recently published Italian protocols for the judgment of suitability for competitive sport (cardiological protocols for judging suitability for competitive sport-COCIS) (Author Anynonymus, 2023) agree with denying the eligibility for sports at risk of trauma if the subject is on OAT (Class III; Level B). According to the Italian protocols, eligibility can be granted to patients with paroxysmal or persistent AF after exclusion of the following conditions: structural heart disease incompatible with sports, major symptoms, presence of a removable trigger (hyperthyroidism, alcohol, drugs, or illicit substances), and a cause-and-effect relationship between sports activity and arrhythmia (Class I, Level C) (Author Anynonymus, 2023). In patients with permanent AF, competitive eligibility may only be granted for dexterity and postural activities, thus avoiding sports with high cardiovascular demands (Class II, Level C) (Author Anynonymus, 2023).

In light of this evidence, participation in contact and collision sports is not recommended, whereas non-contact sports that do not require OAT interruption are considered safe (Maron et al., 2015). Most recommendations have been based on OAT before DOACs. Managing antithrombotic therapy in athletes requires personalized approaches that balance the risks and benefits of the therapy according to the underlying disease and specific sport while considering a potential tailored approach (Delise et al., 2021). DOACs introduce the possibility of exploiting a “therapeutic window” outside of which when the anticoagulant effect is over or considered minimal, even some contact sports could be allowed in appropriate situations (Browne et al., 2018). The shift away from a restrictive approach allows for greater autonomy in prescriptions by incorporating patient preferences into a risk/benefit balance.

For the treatment of deep vein thrombosis (DVT), Berkowitz and Moll (2017) conducted a pharmacokinetic and pharmacodynamic study suggesting that the bleeding risk of athletes treated with DOACs can be minimized by administering DOACs, and interval measurements of the plasma drug concentration are taken over the following 24 h. Thus, by obtaining multiple plasma drug levels during this time, the elimination half-life of the drug can be determined, and the plasma drug concentration correlated with the minimal bleeding risk can be identified. Following the Berkowitz approach, at the end of an athletic competition, when the risk of trauma or bleeding normalizes, a single dose of medication can be administered with rapid onset of the anticoagulant effect (Berkowitz and Moll, 2017).

In a study of DVT, Moll et al. (2018) emphasized on a lack of scientific data about what drug level or physical activity is safe and does not significantly increase the major bleeding risk. The determination of what constitutes a safe DOAC drug level remains empirical. Moreover, for each DOAC, the plasma concentration and half-life have a large interindividual range (Samuelson et al., 2017), and intra-individual fluctuations have been reported, specifically for dabigatran (Chan et al., 2015). All these studies (Chan et al., 2015; Berkowitz and Moll, 2017; Samuelson et al., 2017; Moll et al., 2018) highlight the importance of an individualized pharmacokinetic–pharmacodynamic study, which would require the collection of several blood samples during the day from athletes simulating competition schedules. Residual rivaroxaban and apixaban levels of <30 ng/mL are considered potentially safe for athletes engaged in collision and contact sports. Moreover, an intermittent dosing strategy has been suggested for long-term management of athletes with a history of DVT and the need for OAT, allowing them to return to full athletic activity by discontinuing the drug for some time before engaging in athletic activities that have a risk of causing bleeding and then resuming OAT immediately afterward, if no significant trauma occurs (Moll et al., 2018).

In these cases, when exercise or sports activity is granted to patients on OAT, completing forms could allow better organization of peri-sports activity therapy and monitor the athlete’s health status. Furthermore, written forms legally protect physicians who prescribe OAT, especially when athletes engage in competitive sports. The proposed forms are as follows: an “*athlete return to play acknowledgment of medical condition, potential injury, and informed consent form*;*”* a “*DOAC treatment plan,”* which serves as an overview of care for the athlete and is useful as a therapy reminder in case of any domestic, bleeding, or ischemic events; a “*DOAC drug intake schedule”* for each week, ensuring the last DOAC drug intake before sport practice, especially when significant contact/collision might occur; and “*post-contact athletic participation documentation”* that should be completed by the athlete after the contact sporting activity. The athletic trainer can review the completed forms and notify the physician about any events. In case of a traumatic event, the physician can evaluate the athlete and utilize the *“team physician evaluation for post contact athletic participation risk”* form. If the athlete experiences no significant trauma, the regular DOAC regimen can be resumed (Author Anynonymus, 2023).

Another possible strategy was proposed by Sanna et al. (2018) in their detailed overview of AF in athletes and OAT. They stated that DOACs should be considered the first choice in athletes with AF and an indication for OAT, considering their advantages (no need for INR monitoring, minor drug and food interactions, and potential early cardioversion), thus improving treatment compliance in this group. Furthermore, they shed light on controversial issues, such as the decision to decoagulate athletes with CHA_2_DS_2_-VASc scores of 1 (beyond sex), which is the most common condition encountered in athletic populations due to the low cardiovascular risk profile (i.e., frequently, only hypertension is present).

In conclusion, the guideline recommendations for OAT in athletes only reflect expert consensus and common-sense considerations; therefore, data on therapeutic management in this particular field are still lacking.

## 5 Bleeding management with DOACs

Bleeding events during OAT therapy must be managed according to their severity (Chao et al., 2015). In cases of mild or minor bleeding, delayed or temporary drug discontinuation combined with local hemostatic therapy may be sufficient. If major bleeding occurs, additional measures must be considered, such as administering activated charcoal within 2 h after ingestion, blood derivatives, tranexamic acid, platelet concentration if the platelet count is < 60,000/dL, desmopressin for coagulopathy, hemodialysis, recombinant factor VIIa, and hemodynamic support (Ferri and Corsini, 2015; Romano et al., 2020; Biswas et al., 2023). However, few studies and clinical trials have demonstrated the reversal effects of these efforts in patients with DOAC-associated bleeding (Butler et al., 1993; Wolzt et al., 2004; Pragst et al., 2012; Shih and Crowther, 2016; Tummala et al., 2016; Levy et al., 2018; Hartig et al., 2021). Specific reversal agents that antagonize DOAC activity are useful only in major bleeding occurrences for hospital use. The currently approved antidotes are idarucizumab and andexanet alfa (Biswas et al., 2023).

Another antidote with polyvalent action against direct factor Xa inhibitors, direct thrombin inhibitors, and indirect factor Xa inhibitors (heparins and fondaparinux) is aripazine (PER-977, ciraparantag), an investigational drug that has not been approved by the FDA.

## 6 Discussion

The main problem for patients using OAT who wish to start or continue sports activities is the possibility of traumatic events. Although participation in non-contact sports without the interruption of OAT is considered safe, as previously reported, national and international guidelines are unanimous that participation in limited contact, contact, and collision sports is not recommended (Pelliccia et al., 2005b; Hein et al., 2006; Maron et al., 2015). Removing athletes from play who are engaged in contact and collision sports and have indications for long-term anticoagulation can end their career with potentially impactful psychological and financial consequences.

However, since most recommendations reported in the guidelines are derived from anticoagulant therapy management prior to the introduction of DOACs in clinical practice, the latter, given its pharmacokinetic and pharmacodynamic properties, is a possible resource that could allow the continuation of physical activity without interruption of anticoagulant therapy. A “tailored” strategy has been proposed. To do this, we must first distinguish the cases in which OAT is used to treat an existing thromboembolic condition (such as deep vein thrombosis, pulmonary embolism, and thrombosis in the left atrium) from the cases in which it is used as prophylaxis. In the first case, sporting activity which entails an increase in bleeding risk should be proscribed, as it is absolutely incompatible with the discontinuation of anticoagulant therapy given the high embolic risk. In the case of prophylaxis, however, DOACs could possibility be implemented with “therapeutic windows,” (Berkowitz and Moll, 2017; Moll et al., 2018) outside which, when the anticoagulant effect is finished or minimal, the sporting activity that involves a variable degree of contact could be practiced safely. In this setting, DOACs with lower half-lives or those with twice-daily administration may be useful. The availability of an antidote that rapidly reverses anticoagulant effects can represent an additional advantage and selection criterion.

Several practical aspects should be considered when prescribing DOACs to athletes. First, the selection of a specific DOAC should be based on factors such as renal function, potential drug interactions, and patient preference. DOAC dosing in athletes generally follows standard recommendations, although adjustments may be necessary in specific situations. An alternative management approach based on short-term intermittent interruption of anticoagulation has been proposed in these situations, taking advantage of the “fast on/fast off” characteristics of the DOACs (Moll et al., 2018). DOACs have predictable pharmacokinetics, obviating the need for regular monitoring, as required using warfarin. However, monitoring their blood levels may be helpful in emergency and periprocedural circumstances and in the management of specific groups of patients, such as athletes. DOAC anticoagulant activity can be measured using specific coagulometers developed for the quantification of DOAC plasma levels within 30 min (Douxfils et al., 2012a; Douxfils et al., 2018; Testa et al., 2018). However, the strategy proposed by Berkowitz et al. (Moll et al., 2018), based on a personalized pharmacokinetic/pharmacodynamic study of DOAC for each athlete, is very difficult to apply for at least two reasons: a high level of compliance is required from the athlete due to the need for numerous blood samples and precise care in drug administration and, above all, it creates further difficulties in identifying the right “bleeding threshold”, namely, the plasma level of each DOAC that carries no significant bleeding risk following trauma. Although DOAC plasma levels, at least for dabigatran and edoxaban, have been correlated with bleeding and thrombotic complications (Testa et al., 2018), the DOAC blood level that correlates with a major bleeding risk that is similar to that of a control population is unknown. Furthermore, this threshold level is influenced by an individual’s propensity to bleed. Routine coagulation tests are generally not accurate for assessing DOAC effects and cannot be used to measure anticoagulant activity or provide information on adherence to treatment. The effect of direct Xa inhibitors on PT is highly dependent on the PT reagent used. Therefore, normal PT does not necessarily exclude the therapeutic levels of a particular DOAC. Similarly, point-of-care INR devices developed to monitor coumarin derivatives do not accurately reflect the anticoagulant status of patients treated with DOACs. However, a normal aPTT excludes supratherapeutic levels in dabigatran-treated patients (Douxfils et al., 2012b; van Ryn et al., 2012; Harenberg et al., 2019).

The intermittent strategy, which is based on an empirical approach, appears simpler to implement. DOACs with a shorter half-life or twice daily administration are preferred, and the availability of an antidote represents an additional advantage and selection criterion. Indicative suggestions about safe blood DOAC levels in cases of trauma are taken from guidelines regarding perioperative anticoagulation interruption (Berkowitz and Moll, 2017; Testa et al., 2018; Romano et al., 2020; Douketis et al., 2022; Halvorsen et al., 2022). This intermittent DOAC administration could be safer in subjects who perform collision sports activities occasionally during their leisure time, less suitable for athletes with regular (2–3 times per week) workouts, and not suitable for those who perform daily activities (Romano et al., 2020). Another issue is the appropriate timing for resumption of anticoagulants after play. Based on whether a trauma has occurred, restarting medication within 1–2 h after an uncomplicated sporting event is likely to be safe; however, if trauma is sustained, delayed reinitiation of therapy may be necessary. In the absence of established guidelines, decisions are made on a case-by-case basis with expert guidance after careful consideration of specific circumstances (Berkowitz and Moll, 2017). Specific forms should be completed by the athlete and prescriber to better monitor therapy administration and the athlete’s health status and to legally protect the prescriber. Athletes should maintain regular follow-ups with healthcare providers to assess medication adherence, evaluate renal function, and address any concerns. Regarding the DOAC choice, some post-marketing investigations have compared adherence to treatments with different drugs. Apixaban shows the best adherence, and dabigatran shows the worst adherence because of its capsule formulation, which can cause dyspepsia (Claxton et al., 2001; Desai et al., 2013; Pan et al., 2014). However, the data are discordant. In a review of 76 studies, compliance with single and double administration was not significantly different (Claxton et al., 2001). Athlete education is essential to ensure compliance with medication regimens and discuss the potential side effects, drug interactions, and bleeding management strategies.

## 7 Shared decision-making and collaboration

A tailored approach could represent a solution for athletes who are unwilling to renounce physical activity or for whom suspension has larger economic and psychological implications. However, those who authorize sports activity for athletes on anticoagulant therapy, with schemes that provide a discontinuous intake which is not authorized by the guidelines, are exposed to medical–legal problems if bleeding or thromboembolic events occur. Another issue is the decision to initiate OAT in athletes with AF and CHA2DS2-VASc scores of 1, regardless of sex. Guidelines provide different recommendations for patients for whom it is important to have additional information about the rate of thromboembolic events and trauma-related problems during physical activities to make a decision that can guarantee safe sports activity. Sanna et al. (2018) supported the European approach, arguing that the score does not include other possible risk factors causing a high thromboembolic risk, such as impaired renal function, obstructive sleep apnea, and echocardiographic, biochemical, or coagulation parameters, which can guide the choice of anticoagulation in unclear conditions, such as athletes with AF and CHA2DS2-VASc scores of 1, regardless of sex, and especially those with specific triggers. In athletes with AF, shared decision-making, together with the athlete’s awareness of the pathology and importance of correct therapy, is crucial for optimizing OAT management. Collaboration among cardiologists, sports medicine physicians, and athletes is necessary to develop personalized treatment plans.

## 8 Conclusion

The preventive and therapeutic effects of physical activity are well-established. However, the spread of physical exercise, even among the elderly, has led to an increase in the prevalence of athletes affected by cardiovascular diseases, frequently AF, while on OAT. A severe approach, i.e., denying the possibility of practicing sports to all subjects on OAT, could be counterproductive. Physical activities with a low risk of trauma must always be recommended as a preventive strategy for cardiovascular diseases, whereas activities with a high risk of trauma should be denied for patients on OAT. The optimal management of OAT for patients practicing sports is unclear, and robust evidence is lacking. Recent opinions suggest that DOACs are preferred because of their advantages over coumarin derivatives, such as the lack of the need for INR monitoring, minor drug and food interactions, and more predictable pharmacokinetic effects that can make the eventuality of trauma more manageable. Furthermore, the pharmacological profile of DOACs offers theoretical solutions for overcoming the increased risk of bleeding in these patients. For example, drugs with lower half-lives can allow sports at risk of trauma within the “therapeutic window,” when the estimated blood concentration of the drug is minimal. Further studies to manage OAT in athletes, minimize the bleeding risk, and allow them to safely continue their career are needed; individual clinical evaluations should consider all risks and benefits related to doing or not doing a sports activity, especially in borderline categories such as CHA2DS2-VASc scores of 1 for male and 2 for female athletes. For this purpose, registers containing data on patients practicing non-competitive sports activities who take OAT for AF can be useful for evaluating the incidence of bleeding and embolic events and drawing conclusions on the risk/benefit ratio in these borderline categories. Appropriate patient selection, dosing, adherence monitoring, and shared decision-making are essential for achieving optimal outcomes.
